# A New Phenothiazine-Based Fluorescent Sensor for Detection of Cyanide

**DOI:** 10.3390/bios14010051

**Published:** 2024-01-18

**Authors:** Yulei Li, Chen Zhou, Jiaxin Li, Jing Sun

**Affiliations:** School of Chemistry & Environmental Engineering, Jilin Provincial International Joint Research Center of Photo-Functional Materials and Chemistry, Changchun University of Science and Technology, Changchun 130022, China

**Keywords:** cyanide, fluorescent sensor, phenothiazine

## Abstract

A new fluorescent sensor for the detection of CN^−^ was developed based on the conjugation of phenothiazine fluorophore and benzofuran unit. By the nucleophilic attacking of CN^−^ to the fluoroacetylamino group in the sensor, the additional reaction of CN^−^ and carbonyl group induced the ICT (intramolecular charge transfer) effect in the molecule and caused the fluorescence quenching sensor. The titration experiments show that the sensor has good sensitivity, selectivity and quick response for CN^−^. In addition, the fluorescent detection of CN^−^ in the living cell and zebrafish experiments demonstrated the value of the sensor in tracing the CN^−^ in biological systems.

## 1. Introduction

Cyanide is a highly toxic chemical that is very common in nature; it can be created by the secretion of fungi and algae and also exists in various foods and fruits [1,2]. Although cyanide exists widely, the direct harm to human is not serious due to the low concentration of natural cyanide. With the deepening of social industrialization, a large amount of cyanide waste liquid is produced in the process of metallurgy and fiber production; therefore, cyanide pollution in the surrounding environment is also increasing, even threatening human health [3,4]. Cyanide can be absorbed by the human body in many ways, such as through skin absorption and the respiratory tract. CN^−^ in the human body would prevent Fe^3+^ from being reduced to Fe^2+^, which inhibits the transmission of electrons and affects cell respiration, and then damages the central nervous system, which is most sensitive to hypoxia, causing vomiting, coma and even suffocation [5,6,7]. Research shows that the lethal concentration of cyanide to the human body is only 0.5–3.5 mg/kg, so CN^−^ detection is of great significance in many research fields and it is urgent to develop convenient and effective CN^−^ detection methods [8,9,10,11]. Fluorescence sensors, as a new detection method, have been widely used in the detection of CN^−^ because of their good sensitivity, selectivity and rapid response [12,13,14,15,16,17,18,19]. More and more attention has been paid to the field of fluorescence sensing of CN^−^ in recent years, and many sensing mechanisms have been continuously developed, which are mainly divided into the following types: (1) Hydrogen bond-acting CN^−^ sensor: this type of CN^−^ fluorescent sensors generally has a fast response time, but hydrogen bonding construction is very affected by the acidity and alkalinity in the environment, and some basic anions such as F- have obvious interference for detection [20,21,22,23]; (2) Deprotonated CN^−^ sensor: the response time of CN^−^ fluorescent sensors with a deprotonation mechanism is also short, and compared with the hydrogen bond-acting CN^−^ sensor, it provides better selectivity [24]; (3) Coordination-acting CN^−^ sensor: this type of sensor, formed by the coordination of molecules with metals, is very rich in origin. It can be easily realized by adjusting the spatial configuration and coordination mechanism, and it usually possesses good water solubility, which provides great convenience in the practical application; however, the synthesis process, stability, toxicity and complexation ability of complexes also limit the development and application of such sensors [25,26,27,28,29]; (4) Chemical reaction-based addition CN^−^ sensor: the electron-rich nucleophilic offensive CN^−^ fluorescent sensors have excellent sensing performance. This type of compound not only has good selectivity and sensitivity, but is also accompanied by significant spectral changes. The synthesis process is relatively simple and convenient. It roughly classifies these types of sensors into the following types: C = C bond nucleophilic addition, C = O bond nucleophilic addition, C = N bond nucleophilic addition and nucleophilic addition to electron-deficient compounds. In general, chemical reaction-based addition CN^−^ sensors have attracted much attention due to their excellent performance and have become the fastest developing type of CN^−^ sensors, but there are also some shortcomings in itself, such as slow response time; thus, researchers have been continuously exploring and developing to solve these problems [30,31,32,33,34,35,36,37,38,39].

The near-infrared fluorescent group phenothiazine is widely used in the design and synthesis of fluorescent probes due to its large Stokes shift, long emission wave, and good biological activity [40,41]. Phenylthiazide has a nonplanar “butterfly” structure and is often used as a near-infrared fluorescent dye with a red shift in fluorescence spectra due to its unique intramolecular charge transfer mechanism [42,43,44,45].

In this research, a nucleophilic addition CN^−^ sensor was designed and prepared; it comprises a phenothiazine fluorophore and CN^—^specific addition sites. The nucleophilic attack of CN^−^ to the carbonyl group in the sensor induced the intramolecular charge transfer and inhibited the fluorescence release of phenothiazine. This sensor not only possesses good selectivity and sensitivity but also has fast response time, thus providing more practical applications than the same type of sensors.

## 2. Materials and Methods

### 2.1. Materials

All the chemical reagents and solvents used for synthesis were obtained from commercial suppliers and used without further purification. The solvent for spectra detection was HPLC reagent without fluorescent impurity. Solutions of different ions (CN^−^, NO_3_^−^, Cr_2_O_7_^2−^, BrO_3_^−^, H_2_PO_4_^−^, HPO_4_^2−^, C_2_O_4_^2−^, CrO_4_^2−^, F^−^, I^−^, Na^+^, Zn^2+^, Pb^2+^, Mn^2+^, Mg^2+^, Hg^2+^, Fe^3+^, Co^2+^, Cd^2+^, Ba^2+^) in titration experiments were from NaNO_3_, K_2_Cr_2_O_7_^2−^, NaBrO_3_, NaH_2_PO_4_, Na_2_HPO_4_, C_4_H_12_FN, Na_2_C_2_O_4_, NaCrO_4_·4H_2_O C_4_H_12_FN, C_4_H_12_IN, NaCl, ZnCl_2_, Pb(NO_3_)_2,_ Mn(NO_3_)_2_·6H_2_O, MgCl_2_, Hg(NO_3_)_2_, FeCl_3_, CaCl_2_, CdCl_2_ and BaCl_2_, and they were dissolved in HEPES-NaOH buffer solution at pH 7.4. The stock solution of the sensor was prepared in ethanol. The test samples were prepared by adding accurate amounts of ions stock into the corresponding concentration solution of the sensor [v(ethanol)/v(H_2_O) = 1:1, pH = 7.4]. For fluorometric analysis, the excitation wavelength was set as 375 nm, and the emission wavelength was collected from 400 to 550 nm; both the excitation and emission slit widths were set as 2.5 nm and 2.5 nm, respectively.

### 2.2. Characterization

The resultant sensor 1 was fully characterized by using standard spectroscopic techniques such as ^1^ H NMR and HRMS spectra (Figures S1 and S2). NMR spectra were taken on a Varian mercury-300 spectrometer at an operating frequency of 300 MHz for ^1^H NMR with TMS as an internal standard and DMSO-*d_6_* as solvent. High-resolution mass spectra (HRMS) were recorded on an Agilent (Santa Clara, CA, USA) 1290- micro TOF QII. The UV–Vis absorption spectra were measured using a Shimadzu (Kyoto, Japan) UV-2600. The fluorescence spectra measurements were performed on a Hitachi (Tokyo, Japan) F-4500 spectrofluorimeter. The pH measurements were made with Metteler–Toledo Instruments (Columbus, Ohio, USA) DELTE 320 pH. The cell imaging experiments employed HL-7702 cells (normal liver cells); the live cells were first incubated with sensor solution (30 μM) for 30 min at 37 °C in a 5% CO_2_ atmosphere, then washed with phosphate-buffered saline (PBS, PH = 7.4) for three times and CN^−^ solution (30 μM) was induced into the system for 30 min. The fluorescence cell images were collected by an inverted fluorescence microscope (Olympus (Tokyo, Japan) IX-70) with a digital camera (Olympus, c-5050). The average fluorescence intensity of single cells was analyzed from ImageJ (v1.54).

### 2.3. Synthesis of Sensor 1

**Scheme 1:**
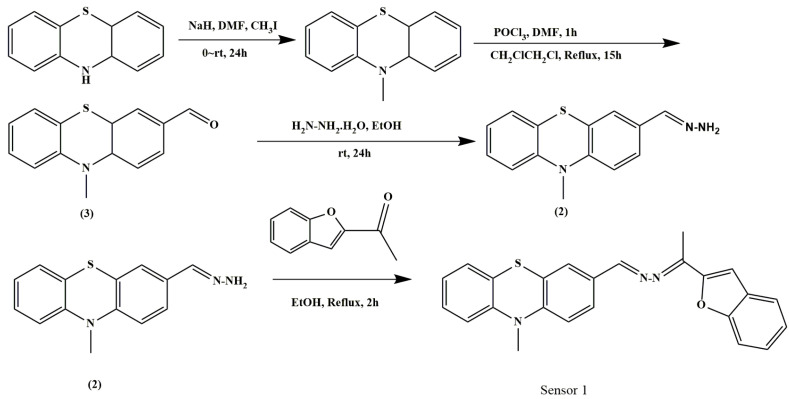
Synthesis of sensor 1.

The synthesis route is shown in Scheme 1. Phenothiazine (4.0 g, 19 mmol) and NaH (0.1 g, 4 mmol) were dissolved in 20 mL DMF and stirred for 0.5 h, and then iodomethane (2.7 g, 19 mmol) was added and stirred for 24 h at room temperature. Afterward, under 0 °C ice bath conditions, phosphorus oxychloride (5.8 g, 38 mmol) was added to the product of the previous step and stirring was continued for two hours. Then, 20 mL 1, 2-dichloroethane was added dropwise and stirring was continued for one hour under 0 °C conditions; the reaction was then heated to 95 °C for 15 h. After the reaction, the solution was cooled to room temperature, extracted three times with deionized water and dichloromethane and the extracted organic phase was dried with anhydrous magnesium sulfate for 12 h. Then, the solvent was evaporated by a rotary evaporator to obtain a brown solid. The crude product was separated and purified by column chromatography to obtain approximately 4.8 g of compound 3, yielding 38% (eluent: dichloromethane/ethanol = 15:1). Compound 3 (2.0 g, 8 mmol) was dissolved in 20 mL ethanol and excess hydrazine hydrate (0.6 g, 12 mmol) was added dropwise and stirred for 24 h at room temperature. A yellow–green precipitate was produced after the reaction, the precipitate was filtered and washed with ethanol for three times and the yellow solid crude product was collected after drying. The crude product was further separated and purified using column chromatography to obtain approximately 1.9 g of compound 2, yielding 73% (eluent: dichloromethane/ethanol = 20/1) [46]. Compound 2 (1.5 g, 6 mmol) and 2-acetylbenzofuran (0.9, 6 mmol) were dissolved in 20 mL anhydrous ethanol and stirred continuously for 2 h at reflux temperature. After the reaction was completed, it was cooled to room temperature and the solvent was evaporated to obtain a yellow solid. The crude product was separated and purified by column chromatography (eluent: dichloromethane) to obtain approximately 1.6 g of final product sensor 1, yielding 67%. ^1^H NMR (300 MHz DMSO-*d_6_*, 25 °C, TMS), δ 2.90(s, 3H), 3.38(s, 3H), 6.99(m, *J* = 12.0, *J* = 9.0 Hz, 1H), 7.04(m, *J* = 4.8, *J* = 6.0 Hz, 1H), 7.08(m, *J* = 3.0, *J* = 2.4 Hz, 2H), 7.22(m, *J* = 6.0, *J* = 5.4 Hz, 2H), 7.41(d, *J* = 3.0 Hz, 2H), 7.53(m, *J* = 9.0, *J* = 6.0 Hz, 1H), 7.56(m, *J* = 3.8, *J* = 4.5 Hz, 2H), 7.61(d, *J* = 4.2, 1H), 8.43(s, 1H). LC-MS. calcd. [(M+H)]^+^ m/z for: C_24_H_19_N_3_OS = 397.13, found [(M+H)]+ m/z = 398.5.

## 3. Results

### 3.1. Absorption Spectral Response

**Figure 1:**
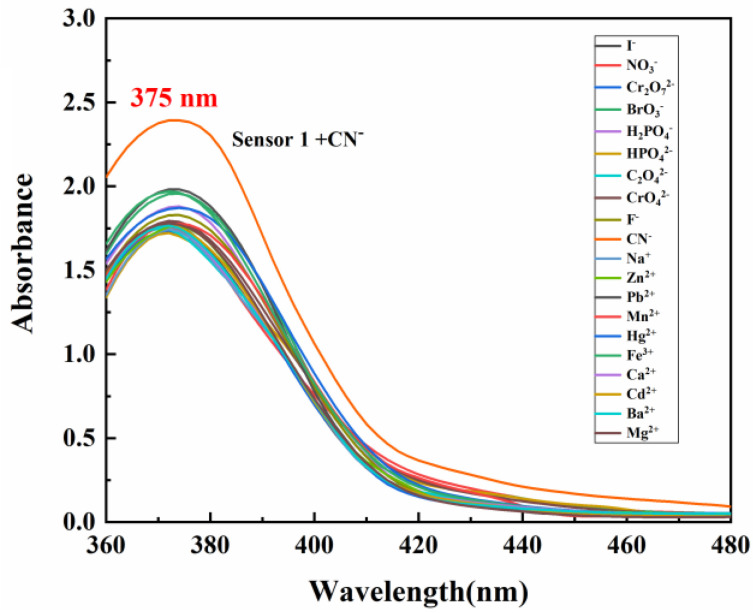
UV-vis absorption response of sensor 1 (0.5 mM) in different ions in HEPES-NaOH buffer (pH = 7.4, 10 mM)−ethanol (1/1, *v*/*v*).

The changes of the UV–vis spectra for sensor 1 in different ions were investigated by titration experiments. The testing system utilized HEPES-NaOH buffer (pH = 7.4, 10 mM)–ethanol (1/1, *v*/*v*) as the solvent; the test temperature was set as 25 °C. Sensor 1 was prepared into ethanol solution with a concentration of 5 × 10^−4^ mol/L, and different kinds of anions and cations (CN^−^, NO_3_^−^, Cr_2_O_7_^2−^, BrO_3_^−^, H_2_PO_4_^−^, HPO_4_^2−^, C_2_O_4_^2−^, CrO_4_^2−^, F^−^, I^−^, Na^+^, Zn^2+^, Pb^2+^, Mn^2+^, Mg^2+^, Hg^2+^, Fe^3+^, Ca^2+^, Cd^2+^, Ba^2+^) at the same concentration and volume were added to test the UV-Vis absorption spectrum in the range of 370~580 nm, as shown in Figure 1. The maximum absorption peak was observed at 390 nm, and the addition of different ions did not have a significant effect on the UV-Vis absorption spectrum of Sensor 1, so 390 nm was used as the excitation wavelength for subsequent fluorescence emission spectrometry.

### 3.2. Fluorescence Spectral Response

**Figure 2:**
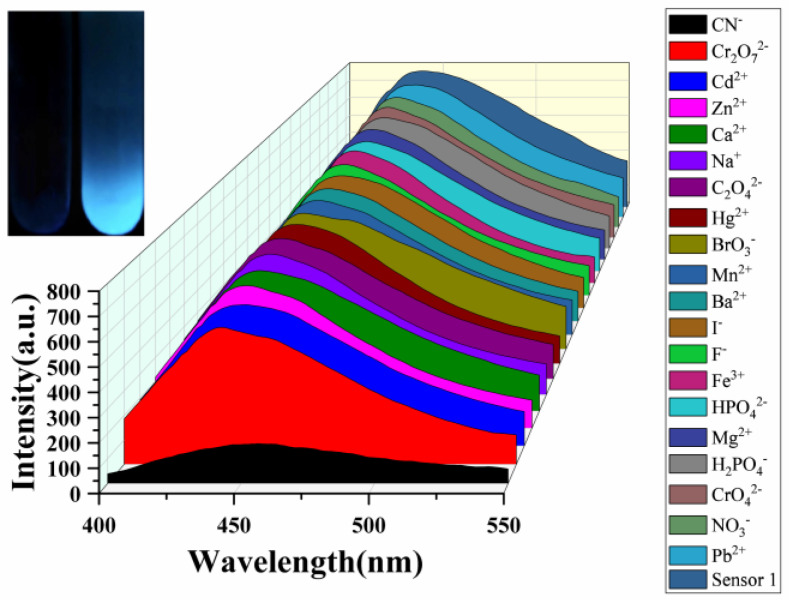
Fluorescence response of sensor 1 (0.5 mM) in different ions in HEPES-NaOH buffer (pH = 7.4, 10 mM)−ethanol (1/1, *v*/*v*), λ_ex_ = 350 nm, slits: 2.5 nm/2.5 nm.

**Figure 3:**
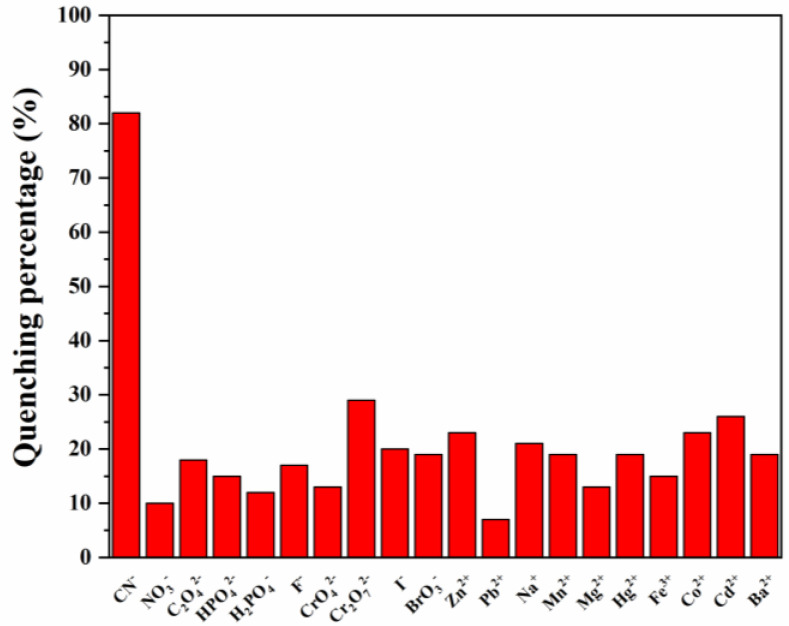
Characterization of fluorescence quenching of sensor 1 in different ions at 445 nm in the fluorescence emission spectrum.

Selectivity is an important indicator for evaluating a sensor. The fluorescence response of sensor 1 among common ions was investigated. The testing system utilized HEPES-NaOH buffer (pH = 7.4, 10 mM)–ethanol (1/1, *v*/*v*) as the solvent; the test temperature was set as 25 °C. Sensor 1 was prepared into ethanol solution with a concentration of 5 × 10^−4^ mol/L, and different kinds of anions and cations (CN^−^, NO_3_^−^, Cr_2_O_7_^2−^, BrO_3_^−^, H_2_PO_4_^−^, HPO_4_^2−^, C_2_O_4_^2−^, CrO_4_^2−^, F^−^, I^−^, Na^+^, Zn^2+^, Pb^2+^, Mn^2+^, Mg^2+^, Hg^2+^, Fe^3+^, Ca^2+^, Cd^2+^, Ba^2+^) at the same concentration and volume were added to test the fluorescence emission spectrum, under the condition that the exit and incident slit width are 2.5 nm and the excitation wavelength is 390 nm. As shown in Figure 2, the maximum emission wavelength of the sensor 1 appeared at 445 nm; after the addition of different ions, it was clear to see that only CN^−^ caused significant fluorescence quenching, and NO_3_^−^, Cr_2_O_7_^2−^, BrO_3_^−^, H_2_PO_4_^−^, HPO_4_^2−^, C_2_O_4_^2−^, CrO_4_^2−^, F^−^, I^−^, Na^+^, Zn^2+^, Pb^2+^, Mn^2+^, Mg^2+^, Hg^2+^, Fe^3+^, Ca^2+^, Cd^2+^ and Ba^2+^ did not have obvious effects on sensor 1 under the same conditions, and this phenomenon of fluorescence quenching is very obvious under the irradiation of an ultraviolet lamp. By comparing the fluorescence intensity of sensor 1 at 445 nm after different ion titrations, the quenching degree induced by CN^−^ could reach more than 80% (Figure 3). So, the above results demonstrated that sensor 1 had good selectivity to CN^−^ from common ions.

**Figure 4:**
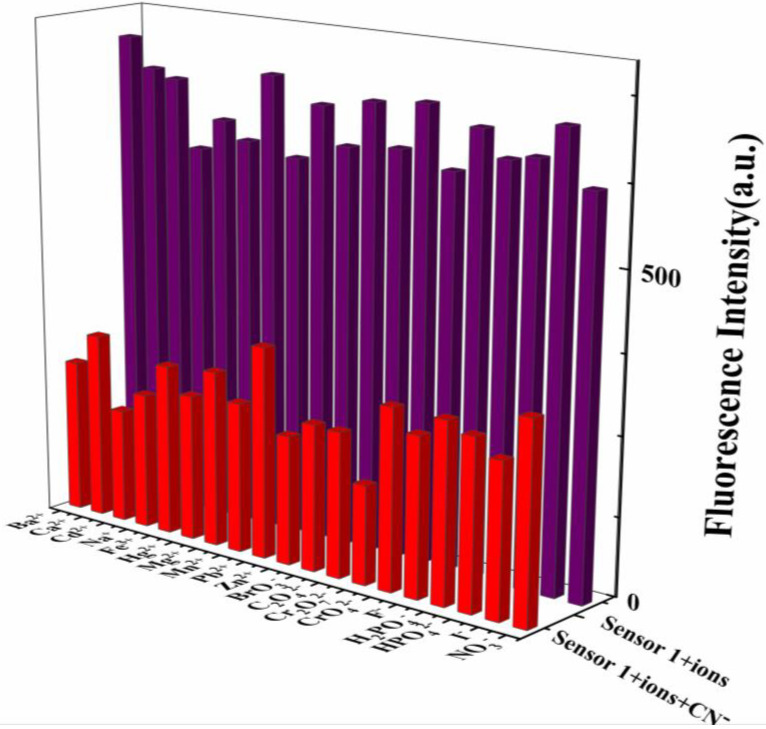
Histograms of fluorescence selective experiments and competitive experiments of sensor 1 in different ions, λ_ex_ = 350 nm, λ_em_ = 445 nm, slits: 2.5 nm/2.5 nm.

Thereafter, competitive experiments were used to verify the specificity of the sensor to CN^−^. First, the fluorescence quenching of sensor 1 was induced by 1.5 equiv CN^−^; after standing for one minute, 10 equiv competitive anions were added, including NO_3_^−^, Cr_2_O_7_^2−^, BrO_3_^−^, H_2_PO_4_^−^, HPO_4_^2−^, C_2_O_4_^2−^, CrO_4_^2−^, F^−^, I^−^, Na^+^, Zn^2+^, Pb^2+^, Mn^2+^, Mg^2+^, Hg^2+^, Fe^3+^, Ca^2+^, Cd^2+^ and Ba^2+^, into the sensor 1-CN^−^ solution, separately. After the full reaction, the fluorescence intensity was measured sequentially with 390 nm as the excitation wavelength, and finally the fluorescence intensity at the maximum emission of 445 nm was taken as a histogram for comparison. As shown in Figure 4, the purple bar represents the fluorescence intensities of selective experiments and the red bar represents the competitive experiments. It is obvious to find that other anions did not interfere with the detection of CN^−^. Consequently, the selective and competitive experimental results show that sensor 1 had good specific recognition ability for CN^−^ and provide a basis for qualitative analysis of CN^−^.

**Figure 5:**
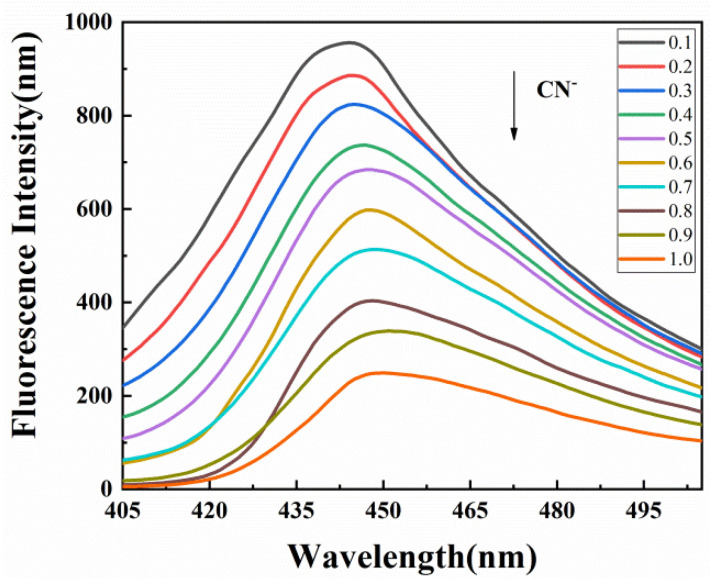
Fluorescence response of sensor 1 (0.5 mM) to CN^−^ in different concentrations (0.05–0.5 mM) in HEPES-NaOH buffer (pH = 7.4, 10 mM)−ethanol (1/1, *v/v*), λ_ex_ = 350 nm, slits: 2.5 nm/2.5 nm.

**Figure 6:**
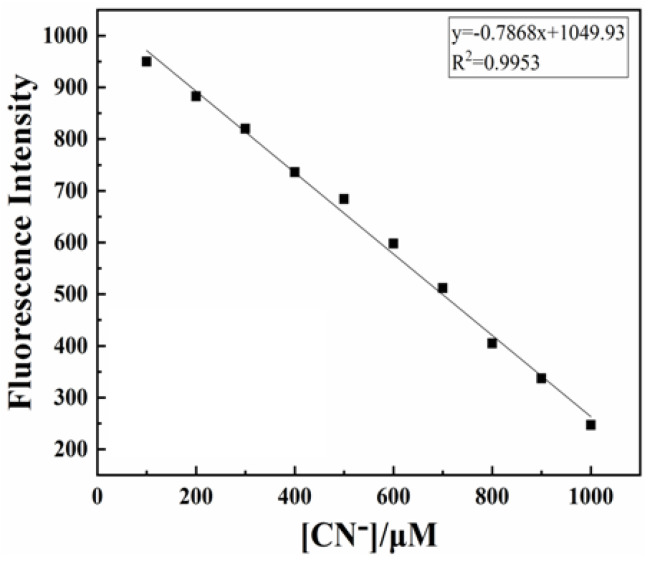
Normalized response of fluorescence signal to CN^−^ in different concentrations.

To detect the sensitivity of sensor 1 to CN^−^, the changes in the fluorescence spectra for sensor 1 in different concentrations of CN^−^ were investigated. The testing system utilized HEPES-NaOH buffer (pH = 7.4, 10 mM)–ethanol (1/1, *v/v*) as the solvent; the test temperature was set as 25 °C. Figure 5 illustrates the fluorescence emission spectra response of sensor 1 in different CN^−^ concentrations (λ_ex_ = 390 nm). As depicted in Figure 5, the fluorescence intensity at 445 nm decreased gradually with the increase in CN^−^ concentrations in the sensor 1 solution. Moreover, such reduction in fluorescence emission behavior of sensor 1 toward CN^−^ showed a good linear relationship. Figure 6 illustrates the plots of the fluorescence intensities of sensor 1 solution at 445 nm vs. the concentrations of CN^−^. Remarkably, the fluorescence intensity of sensor 1 varies almost linearly vs. the concentration of CN^−^ in the range of 0.1–1 equiv, with the coefficient R^2^ = 0.9953. This phenomenon illustrated that sensor 1 had a potential application for quantitative determination of CN^−^ concentrations. The detection limit (DL) of sensor 1 toward CN^−^ was determined from the following equation:

(1) $$DL=\frac{K\times Sb_{1}}{S}$$

where K = 2 or 3 (we take 2 in this case); Sb_1_ is the standard deviation of the blank solution; S is the slope of the calibration curve. From this equation, the detection limit of sensor 1 towards CN^−^ was calculated to be 2.0 × 10^−6^ M^−1^. By comparing the published organic fluorescent sensors for the detection of CN^−^, it was found that sensor 1 had a good detection effect on CN^−^.

The binding ability of the sensor to the identified substance often determines the sensing performance. Therefore, the binding constant and binding ratio of sensor 1 to CN^−^ were determined from the Stern–Volmer equation [47]:

(2) $$\mathrm{lg}(\frac{I_{0}-I}{I})=\mathrm{lg}K_{SV}+\mathrm{nlg}(Q)$$

**Figure 7:**
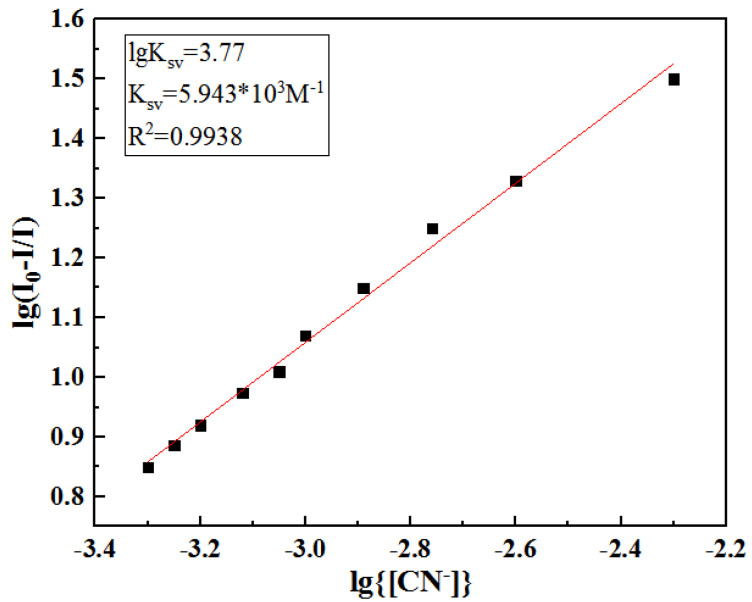
The linear fitting of the fluorescence titration curve of sensor 1.

**Figure 8:**
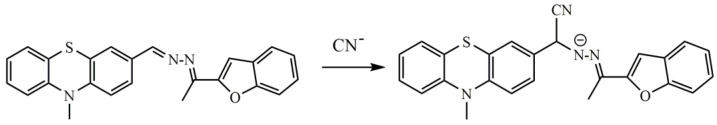
The combined model diagram between sensor 1 and CN^−^.

As illustrated in Figure 7, the corresponding data were substituted into the Stern–Volmer equation. It could be calculated that the complexation constant of sensor 1 with CN^−^ was 5.943 × 10^3^ M^−1^, and the binding ratio was 1:1. Based on the reports in the relevant literature, it was further speculated that a possible binding model between the probe and CN^−^ was shown in Figure 8. Due to the strong nucleophilicity of CN^−^, after binding with sensor 1, a nucleophilic addition reaction occurred between CN^−^ and C = N, producing the ICT effect that suppressed fluorescence release and led to significant fluorescence quenching of sensor 1, achieving the recognition of CN^−^ [48,49]. Therefore, we affirm that sensor 1 belongs to chemical reaction-based addition CN^−^ sensor. Compared with other types of CN^−^ sensor, the electron-rich nucleophilic offensive CN^−^ fluorescent sensors displays good selectivity and sensitivity and avoids interference from F^−^ in the hydrogen-bond-acting CN^−^ sensor. Moreover, the synthesis process is relatively simple and convenient and accompanied by significant spectral changes, so sensor 1 is provided with potential applicability on account of the above advantages.

**Figure 9:**
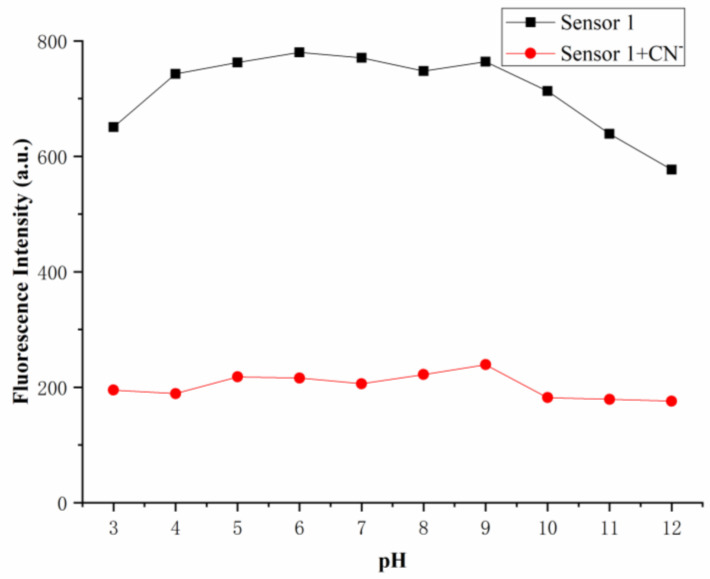
The effect of pH on the fluorescence intensity of sensor 1 and sensor 1- CN^−.^

pH stability is an important indicator for measuring the application value of a sensor. So, the effects of the pH of sensor 1 in the absence and presence of CN^−^ were detected from pH 3.0 to pH 12.0. As illustrated in Figure 9, the fluorescence intensity of sensor 1 decreased slightly under high acidic or alkaline conditions, while it remained relatively stable in the pH range of 4–9. Upon the addition of CN^−^, the fluorescence quenching was very obvious in a different pH range. So, the pH effect on the fluorescence intensity of sensor 1 and sensor 1-CN^−^ proved that sensor 1 was capable of detecting CN^−^ in a wide pH range (4–9). In addition, considering that most samples for CN^−^ analysis were neutral, the media for CN^−^ detection was buffered at pH 7.4.

### 3.3. Practical Applications

**Figure 10:**
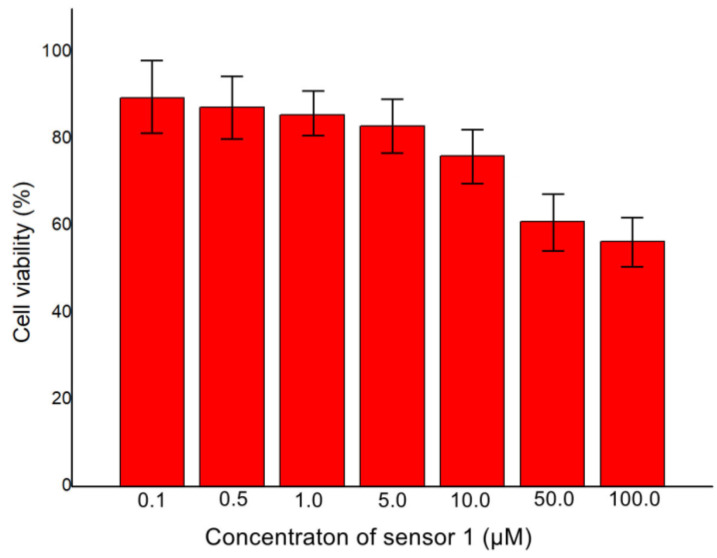
Concentration-dependent cell viability assay.

To verify the potential application of sensor 1 in biological systems, the MTT assay was adopted in HL-7702 cells (normal liver cells) to test the cytotoxicity in different concentrations (100.0, 50.0, 10.0, 5.0, 1.0, 0.5 and 0.1 μM) of sensor 1. As shown in Figure 10, the cells did not exhibit any obvious decrease in cell viability from concentrations of 10.0 to 0.1 µM. However, when the concentration reached 50 um and 100 um, the cell viability slightly decreased, reaching a minimum of 58%, and the IC50 value was ascertained as 195.8 μM. So, the above experimental results demonstrated that sensor 1 has low cytotoxicity. Accordingly, sensor 1 could indeed be used to track CN^−^ in vivo.

**Figure 11:**
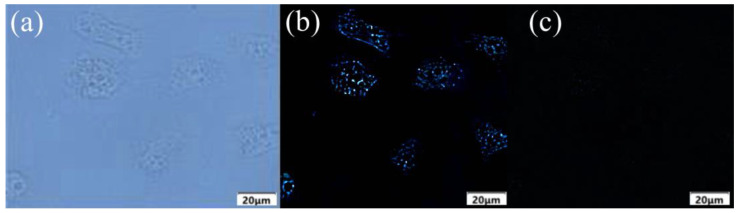
(**a**) Bright field transmission image of HL-7702 cells treated with sensor 1 (30 μM); (**b**) fluorescence imaging of HL-7702 cells treated with sensor 1; (**c**) fluorescence imaging of HL-7702 cells treated with sensor 1-CN^−^ (45 μM).

Therefore, the live cells experiments and live fish experiments were studied to further verify the biocompatibility of sensor 1; the capability of sensor 1 to track CN^−^ was also researched in HL-7702 cells. The live cells were first incubated with sensor 1 solution (30 μM) for 30 min at 37 °C in a 5% CO_2_ atmosphere, then washed with phosphate-buffered saline (PBS, PH = 7.4) for three times and CN^−^ solution (30 μM) was induced into the system for 30 min. As shown in Figure 11a,b, the cells which were only treated with sensor 1 emitted evident fluorescence and still maintained clear contours. The average fluorescence intensity of single cells treated with sensor 1 was 44.018, but after the addition of CN^−^, as illustrated in Figure 11c, the fluorescence in HL-7702 cells quenched obviously, and the average fluorescence intensity of single cells had decreased to 3.675. According to the above images, we proved that sensor 1 could be successfully immersed into living cells and had the ability to trace CN^−^ in biological cells.

**Figure 12:**
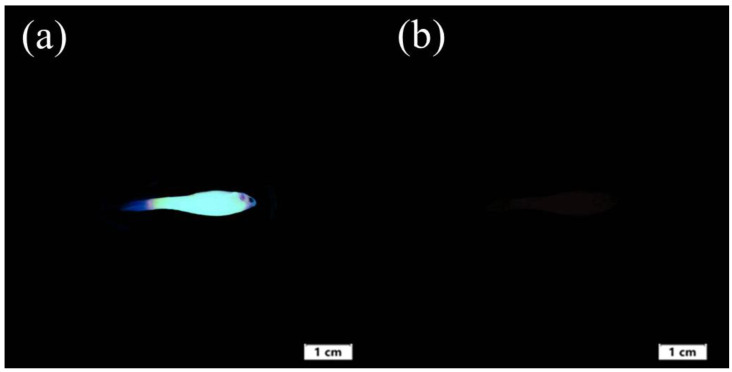
Images of zebrafish under 365 nm UV light; (**a**) zebrafish bred with only sensor 1 (10 μM) for 1 h; (**b**) zebrafish bred with sensor 1 and then bred with CN^−^ (10 μM) for 1 h.

To further explore the capacity of sensor 1 for detecting CN^−^ in live animals, the zebrafish was used as a specimen for a series of experiments. The fish were fed with sensor 1 (10 μM) dissolved in DMSO for 20 min, then washed with phosphate-buffered saline (PBS, pH = 7.4) three times and the fish were treated with CN^−^ solution (10 μM) for 1h. As illustrated in Figure 12a, the fish exposed to sensor 1 displayed apparent obvious fluorescence under 365 nm light, but, in Figure 12b, the zebrafish treated with both sensor 1 and CN^−^ showed no more fluorescence. From the above images, it was clear that sensor 1 was successfully immersed into the zebrafish body, and it was obvious from the fluorescence intensity that the accumulations of sensor 1 were mainly stacked in the gills and abdomen of the fish. Generally speaking, the live cell and animal imaging experiments demonstrated that sensor 1 was membrane permeable and had good biocompatibility; it could be a valuable molecular sensor for detecting CN^−^ in the live system.

## 4. Conclusions

In this research, a new phenothiazine-based fluorescent sensor for the detection of CN^−^ was successfully developed. The introduction of CN^−^ to sensor 1 would induce obvious fluorescence quenching due to the nucleophilic addition reaction between CN^−^ and C = N, which produced the ICT effect that suppressed fluorescence release from sensor 1. The UV-Vis absorption spectrum and fluorescence emission spectrum were used to test the sensitivity and selectivity of this sensor, and the results reflected that the sensor had both the ability of qualitative and quantitative analysis of CN^−^ and the detection limit of CN^−^ can reach 2.0 × 10^−6^ M^−1^. According to the Stern–Volmer equation, it can be inferred that the binding constant of sensor 1 for CN^−^ was calculated to be 5.943 × 10^3^ M^−1^ and the binding ratio between sensor 1 and CN^−^ was 1: 1. In addition, the fluorescent detection of CN^−^ in the living cell and zebrafish experiments expanded the applications of the sensor in biological systems.

## Data Availability

The data presented in this study are available in Supplementary Materials.

## Supplementary Materials

The following supporting information can be downloaded at: https://www.mdpi.com/article/10.3390/bios14010051/s1, Figure S1: ^1^H NMR spectrum of sensor 1; Figure S2: LC-MS of sensor 1.
