# Supplementary material for: A New Phenothiazine-Based Fluorescent Sensor for Detection of Cyanide
Source: Biosensors (Basel). 2024 Jan 18;14(1):51. doi: 10.3390/bios14010051 (PMC10813016; doi:10.3390/bios14010051)
Supplement: Supplementary file 1 [file biosensors-14-00051-s001.zip › biosensors-2791545-supplementary.pdf]

A New Phenothiazine-based Fluorescent Sensor for Detection of Cyanide

Yulei Li [1], Chen Zhou [1,*], Jianxin, Li [1] and Jing Sun [1]

[1] School of Chemistry & Environmental Engineering, Jilin Provincial International Joint Research Center of Photo-functional Materials and Chemistry, Changchun University of Science and Technology, Changchun, 130022, People's Republic of China
Corresponding author: Chen Zhou
E-mail address: zhouchen@cust.edu.cn
Tel: +86431 85583894

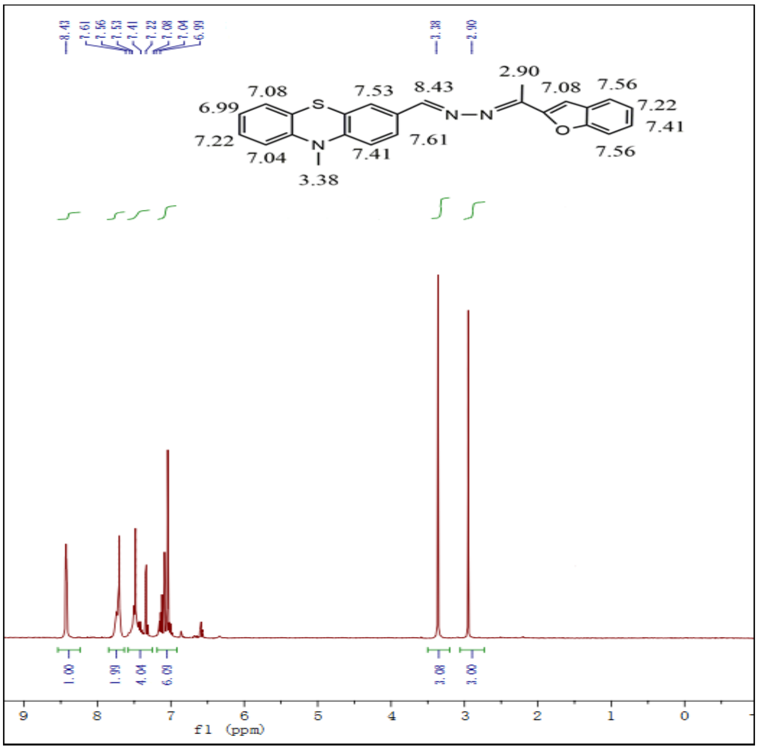

Figure S1. ^{1}H NMR spectrum of sensor 1

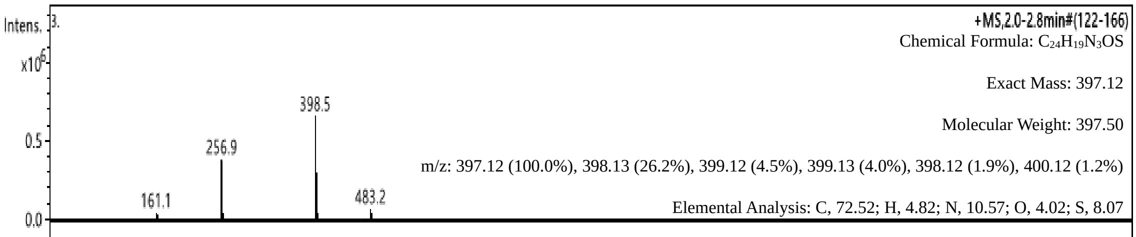

Figure S2. LC-MS of sensor 1
